# Harnessing Aptamers to Overcome Challenges in Gluten Detection

**DOI:** 10.3390/bios6020016

**Published:** 2016-04-20

**Authors:** Rebeca Miranda-Castro, Noemí de-los-Santos-Álvarez, Arturo J. Miranda-Ordieres, María Jesús Lobo-Castañón

**Affiliations:** Departamento de Química-Física y Analítica, Universidad de Oviedo, Julián Clavería, 8, 33006 Oviedo, Spain; mirandarebeca@uniovi.es (R.M.-C.); santosnoemi@uniovi.es (N.S.-Á.); amir@uniovi.es (A.J.M.-O.)

**Keywords:** aptamers, biosensors, celiac disease, gluten, gliadin, prolamins

## Abstract

Celiac disease is a lifelong autoimmune disorder triggered by foods containing gluten, the storage protein in wheat, rye, and barley. The rapidly escalating number of patients diagnosed with this disease poses a great challenge to both food industry and authorities to guarantee food safety for all. Therefore, intensive efforts are being made to establish minimal disease-eliciting doses of gluten and consequently to improve gluten-free labeling. These efforts depend to a high degree on the availability of methods capable of detecting the protein in food samples at levels as low as possible. Current analytical approaches rely on the use of antibodies as selective recognition elements. With limited sensitivity, these methods exhibit some deficiencies that compromise the accuracy of the obtained results. Aptamers provide an ideal alternative for designing biosensors for fast and selective measurement of gluten in foods. This article highlights the challenges in gluten detection, the current status of the use of aptamers for solving this problem, and what remains to be done to move these systems into commercial applications.

## 1. Rationale for Gluten Detection

Gluten, from the Latin word “glue,” is a complex mixture of storage proteins from grains, which are used as ingredients in a wide range of foods. The introduction of gluten in our diet occurred about ten thousand years ago, and only in certain parts of the world. This is a relatively short period in the human history, and it appears that many individuals did not adapt to processing it well and did not develop immunological tolerance to it. In fact, celiac disease (CD), a complex autoimmune disorder with immunological, genetic, and environmental components that is triggered by the ingestion of gluten, is nowadays a common disease affecting one in every hundred individuals in Western populations [1]. The only effective treatment currently available for these patients is a strict, life-long gluten-free diet. Along with CD, there are other gluten-related disorders [2], and a growing number of consumers consider gluten-free products healthy. As a consequence, the production of gluten-free foods has a thriving market, with global sales approaching $2.5 billion (US) in 2010 and an estimated $6.2 billion (US) by 2018 [3].

In order to ensure safety, quality, and fairness of international food trade, the Codex Alimentarius Commission includes cereals containing gluten in the list of foods and ingredients that shall always be declared [4], and establishes that gluten level should not exceed 20 mg/kg or 100 mg/kg for “gluten-free” and “very low gluten content” foods, respectively [5]. Following the Codex Alimentarius recommendations, many countries, including the European Union, Canada, and USA, adopted these threshold values in their legislation. Even if these values have proven to be useful in managing the diet of an important part of celiac patients, there are individuals sensitive to lower gluten intakes. In an attempt to protect a larger number of celiac people, the Spanish Federation of CD Patients Associations (FACE) has its own label that guarantees an amount of gluten below 10 mg/kg (10 ppm). 

To assess compliance with labeling legislation and guarantee the safety of these products, several analytical methods have been developed [6], but, despite major efforts, an accurate quantification of gluten in a variety of food matrices still represents considerable challenges. In fact, current threshold values for labeling are limited by the sensitivity of the analytical methods used to verify the compliance. The lowest gluten content, which is guaranteed by the Spanish label FACE [7] is very close to the limit of quantification provided by the existing analytical methods, but it is not safe enough for some celiac patients. Therefore, the analytical chemists’ community has to confront this problem and to provide extremely sensitive and innovative tools for gluten control in food. Aptamer-based assays can make spectacular contributions to this problem, even beyond the analytical field. The purpose of this paper is to provide an up-to-date overview of the state of gluten detection methods, highlighting major challenges and the recent advances in aptamer-based assays. A global wave of investment in this new technology will open many opportunities in bioanalytical chemistry.

## 2. Requirements and Challenges in Gluten Detection

**Figure 1:**
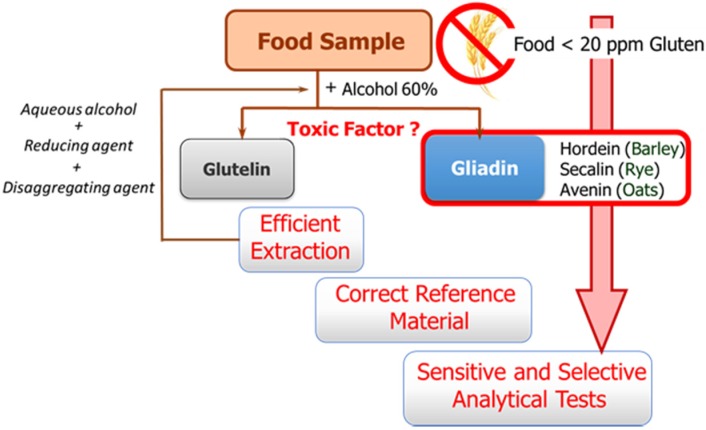
Schematic of the main steps and challenges associated with gluten determination in food samples.

The availability of sensitive and robust analytical methods to quantify gluten in foods is of utmost importance not only to ensure the well-being of gluten sensitive individuals but also for food control authorities and the food industry. The success of the measurement relies on three main factors as illustrated in Figure 1: efficient extraction from both raw and processed foods of the harmful proteins and peptides, the use of a correct standard representative of gluten proteins for calibration, and the sensitivity and selectivity of the selected method.

These three factors are hampered by the structural complexity of gluten. The term gluten encompasses the storage proteins found in the starchy endosperm of various cereals, viz. wheat, barley, rye, their hybrids, and possibly oat. This means that it is a mixture of hundred proteins of intricate composition, which are among the most complex protein networks in nature due to both their varied composition and size, and the variability caused by genotype, growing conditions, and technological processes [8]. Gluten proteins are classically divided into two main fractions according to their solubility in alcohol-water solutions without reduction of disulfide bonds: the soluble prolamins and the insoluble glutelins. Prolamins receive this name because of their high content in the amino acids proline and glutamine, and are named as gliadins (wheat), secalins (rye), hordeins (barley), and avenins (oat), depending on the cereal. Although traditionally only prolamins were considered toxic, there is evidence that glutelins can also trigger an immune response. Their proline-rich nature makes gluten proteins resistant to complete proteolytic degradation by digestive enzymes. Therefore, long peptide fragments survive in the stomach and small intestine after digestion, and these are the true precipitating factor in predisposed individuals [9]. Different active peptides have been identified up to now; their composition, predominated by glutamine and proline, contains hydrophobic amino acid residues such as phenylalanine, tyrosine, and leucine, with many repetitive units varying in length and frequency depending on the origin [9]. Targeting this repetitive oligopeptides will allow for a more accurate assessment of toxicity levels.

Gluten extraction protocols should be unbiased, valid for different matrices, and compatible with the method used for quantification. Although the most commonly used solvent in gluten extraction is aqueous alcohol (60% ethanol or 50% propanol) [6], it can only extract the prolamin fraction from non-processed materials. The calculation of gluten content is in this case performed using the assumption of a 1:1 ratio between prolamins and glutelins. However, differences in the ratio between the two gluten components depending on the variety and species of the cereal may lead to important errors [8]. Food sample processing is another determining factor because it affects the reliability of results by leading to an underestimation of food toxicity in certain situations. Highly processed samples have been subjected to thermal and enzymatic transformations in which the proteins are degraded and hydrolyzed, giving rise to shorter amino acid sequences, which often remain toxic, but they could circumvent certain gluten control tests. Likewise, the heat treatment undergone by foods during manufacturing negatively affects the recovery of gluten proteins, as it leads to the formation of protein aggregates, which are insoluble in the alcohol-water mixtures. Therefore, in thermally processed samples, additional disaggregating and reducing agents such as guanidine chloride and β-mercaptoethanol, capable of breaking the interchain S-S bonds of protein and solubilizing gluten, are necessary. The use of these reagents has led to the extraction solution known as a “cocktail” [10]. However, some quantification assays (immunoassays) are incompatible with the extraction cocktail, because its components can denature the protein receptor. In order to solve this problem, different gluten extraction solutions have been studied [11]. Among them, UPEX (universal prolamin and glutelin extractant solution), containing the reducing agent Tris(2-carboxyethyl)-phosphine and the disaggregating compound N-lauroylsarcosine [12], seems to be compatible with immunochemical quantification methods and improves the extraction of native gluten protein as well as the denatured one. In some particular cases, such as samples with a high amount of fats, additional treatment may be necessary in order to eliminate interferences from the matrix. In any case, it is necessary to verify the compatibility of the extraction process with the selected quantification method.

The second general problem to be addressed is the selection of a correct reference material for calibration. Achieving a standard of gluten has become a tough task, and no certified reference material is indeed available so far. A mixture of 28 European wheat cultivars prepared by the Working Group on Prolamin Analysis and Toxicity, the so-called PWG [13], is the most used material for calibration and validation. Although this is a homogeneous and stable material, it only represents the prolamin fraction of some cereals, and in some cases the use of incurred materials, albeit very costly to obtain, are recommended [6].

Generally, the available methods for gluten analysis in foodstuffs can be merged into two groups: (i) direct ones, sensing immunotoxic proteins and peptides, including immunochemical assays, immunosensors [14] and mass spectrometry analysis [15]; and (ii) indirect ones, based on the detection of the DNA of the gluten-containing cereals, genosensors [16] and PCR [17]. Although DNA analysis is more sensitive than protein analysis, it should be considered as a screening tool to confirm the presence of gluten, and as a complement of the protein-based methods. Moreover, legislation thresholds are issued in terms of gluten concentration. A precise correlation between the DNA copy number and gluten concentration in each matrix would be needed to use DNA-based methods for quantitation purposes. Mass spectrometry analysis is mainly used for the identification of the protein and peptide profiles from different cereals, but it is not considered as suitable for routine analysis because it requires expensive equipment and expertise [6]. Therefore, enzyme-linked immunosorbent assays (ELISAs) are currently the method of choice for gluten determination in food. Several antibodies targeting different fragments of gluten proteins have been obtained [6]. Among them, the monoclonal antibodies, termed Skerrit [18] raised against ω-gliadins, R5 [19] obtained against ω-secalins, and G12 [20] raised against a 33-mer peptide from α2-gliadin, are the most commonly used in commercial ELISA kits. Two different assay formats—sandwich and competitive—are possible. However, methods based on a sandwich assay, where the target is recognized and sandwiched between two antibodies, are not suitable for quantifying gluten in hydrolyzed samples because the presence of protein fragments with more than one binding site is unlikely. This drawback can be overcome by using competitive assays where target does not require multiple epitopes to be quantified [12]. However, these immunoassays are in general not fully compatible with the cocktail extraction solution because their components may denature the protein receptors [21]. Table 1 compiles the main commercial immunoassays for gluten detection in food, comparing their analytical performance. A non-protein receptor for gliadin would thus contribute to the improvement of gluten detection methods. Nucleic acid aptamers, obtained by the *in vitro* selection process SELEX, are a good alternative.

## 3. Selection of Gluten-Binding Aptamers

*In-vitro* selection of aptamers always yields a variety of sequences of higher affinity than the initial pool, which promotes the misconception of a sure and successful procedure. Now it is commonplace that not all targets are equally prone to generate useful aptamers (that is, with an affinity in the low nM or even pM range), and the SELEX success is quantified by some authors to be less than 30% [22]. Unfortunately, there are no general rules to anticipate this behavior. Gluten is one of those elusive targets probably due to its hydrophobicity that does not fit well with the hydrophilic nature of nucleic acids. Though we are aware of several failed SELEX for gluten, we succeeded upon the rational and very careful selection of the specific target and its immobilization strategy [23].

An immobilization-free interaction was discarded to avoid filter partitioning that is labor-intensive and prone to unspecific binding. Likewise, the use of gliadin was also rejected because of their insolubility in aqueous media and difficult immobilization through a covalent (oriented) bond. The immunodominant peptide known as 33-mer [9] is an apolar compound that can be dissolved in water at the concentrations required for SELEX. A recombinant variant with a large spacer of 57 amino acids and a 6-His-tag tail at C-terminal end was selected for immobilization on Ni^2+^-nitrilotriacetic acid magnetic beads (Ni^2+^-NTA MBs). This presentation is advantageous because the long spacer chain minimizes the steric hindrance with the surface during interaction and exposes the target to the bulk solution facilitating the recognition. Negative selections to remove any spacer-binding aptamer are compulsory and were carried out every three rounds of selection. The binding between Ni^2+^ and histidines is strong enough to suffer washing steps but also labile enough to be displaced by a high concentration of a competitor like imidazole, allowing the easy elution of the aptamer-peptide complex for subsequent PCR amplification.

The selection buffer was carefully chosen to have a high ionic strength to minimize unspecific electrostatic interactions if possible when using a mostly apolar target, and 1 µg/mL BSA was added to avoid unspecific adsorption to the surface. A t-RNA was added as a competitor in a ten-fold lower concentration than the DNA library or pool in each round. The stringency was progressively increased by reducing the interaction time from 1 h to 15 min and increasing the washing steps from 2 to 15. The combination of two factors is unusually carried out and could be a relevant factor for the successful selection.

PCR amplification tends to bias the selection toward sequences with weak secondary structures that are easier to amplify. Likewise, the higher the number of cycles, the higher the chances to produce artifacts (mainly primer-dimers) [24]. For those reasons, the number of cycles was kept below 18 cycles. If the recovery was not high enough to initiate the following round, a new PCR was performed using 15 cycles. In all cases, a biotinylated reverse primer was used to allow the separation of the strands prior to the next round of selection. The strand separation was performed by amplicon entrapment on streptavidin-coated magnetic beads and dehybridization in 100 mM NaOH followed by magnetic separation. The supernatant was neutralized with HCl prior to being diluted in the selection buffer. This method does not require the purification of amplicons as exonuclease digestion, minimizing the risks of sequence loss [25].

After ten rounds of selection, a pool of aptamers with significant affinity to the target and negligible to the solid support and spacer was obtained. This was verified by fluorescent binding assays on Ni^2+^-NTA MBs coated by the recombinant 33-mer or the control peptide (the spacer). The aptamer candidates were obtained from each round pool by PCR amplification using a forward primer labeled with a fluorophore (6-carboxyfluorescein) and the biotinylated reverse primer.

Sequences from round 10 were cloned, sequenced, and analyzed for sequence homologies. They were clustered into five families with a diverse degree of homology. Variations of a 12-nt motif were found in four groups with a GTCT core motif shared by all groups, which is an unusual result that supports a strong convergence.

**Figure 2:**
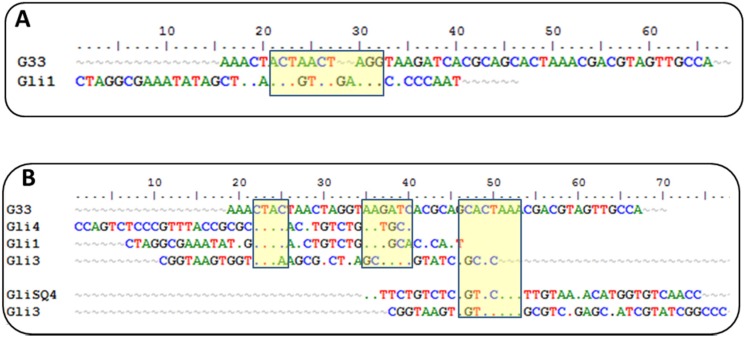
(**A**) Alignment of anti-gliadin G33 and anti-peptide Gli1 aptamers. The 12-nt motif is marked in yellow. (**B**) Global alignments of anti-gliadin G33 and anti-peptides aptamers. The homologous regions are marked in yellow.

Currently, there is another aptamer for gliadin detection, obtained using gliadin as a target for selection [26]. A close inspection of the winning sequences from both SELEX procedures results in some common characteristics: a low GC content and the absence of a strong secondary structure, just the opposite for most aptamers described, indicating that it is not a bias of the procedure but a real evolution for that type of target. This anti-gliadin aptamer has the highest level of homology to the anti-peptide Gli-1 aptamer (the most abundant one) within the 12-nt motif. Additional alignments of 33-mer aptamers to the anti-gliadin aptamer show some minor similarities, with short homology regions (Figure 2). Interestingly, a 9-nt sequence (CGACGTAGT) is shared with an aptamer that was unique in our enriched pool.

Such level of coincidence is striking taking into account the significant differences between both processes. The gliadin aptamer was evolved using Sigma gliadin as a target instead of the accepted “reference” material (PWG). Chemical properties of this gliadin are quite different from PWG (e.g., it cannot be dissolved in alcohol-water mixtures), and it is not representative of wheat genetic variability. The protein was adsorbed on hydrophobic microtiter plates rendering a denatured target with the hydrophilic moieties exposed to the solution. The random orientation of the target along with the lack of negative selections can explain the lack of a consensus sequence among the clones except for only three of them. The stringency was enhanced only by increasing the number of washing steps. On the other hand, the selection buffer also contained BSA, t-RNA, and a high salt concentration but not as high as in our procedure. Eight rounds of selections were finally performed, and SPR was used for enrichment assessment with gliadin covalently immobilized through amine groups on a dextran matrix.

## 4. Aptamer-Based Assays for Gluten Detection

Selecting aptamers as the biomolecular recognition element for developing alternative gluten detection methods relies on various reasons: (i) they are readily obtained by chemical synthesis in a cost-effective way and with high reproducibility; (ii) they show affinities comparable to those of monoclonal antibodies but with higher stability due to their nucleic-acid chemical nature; and (iii) they can be easily combined with different chemical labels/groups that provide flexibility for adaptation to different platforms, taking advantage of existing analytical techniques.

**Figure 3:**
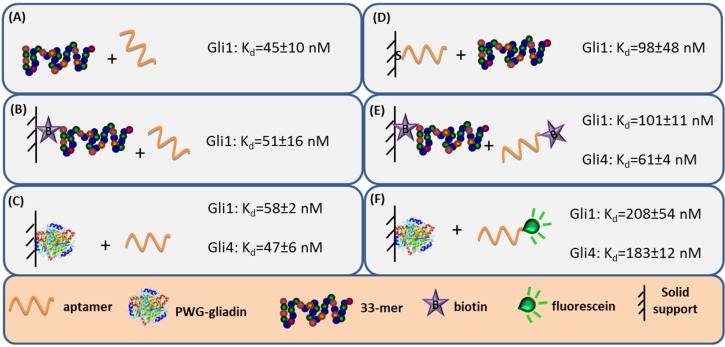
Schematic of the different methods used for evaluating the aptamer binding affinity and comparison of the binding constants obtained for the interaction between aptamers and 33-mer peptide or PWG. Constants evaluated by (**A**) isothermal titration calorimetry (ITC); (**B**) surface plasmon resonance spectroscopy (SPR); (**C**) ICP-MS; (**D**) faradaic impedance spectroscopy (FIS); (**E**) chronoamperometry; and (**F**) fluorescence.

Aptamer binding affinity is the main criteria to select the receptor. Hence, the capability to accurately measure binding interaction is very important and numerous analytical methods have been used to characterize the anti-gliadin aptamers [27]. Label-free techniques such as Surface Plasmon Resonance (SPR) spectroscopy and Faradaic Impedance Spectroscopy (FIS) are especially suitable to evaluate the binding affinity when one of the interacting partners is immobilized. Considering the homogeneous 33-mer peptide-aptamer interaction, characterized by isothermal titration calorimetry, as a reference (Figure 3, panel A), a slight decrease in the aptamers affinity was recorded when 33-mer peptide was anchored to an SPR sensor (Figure 3, panel B). Meanwhile, the PWG-gliadin attachment onto a solid support enlightened the presence of multiple sites for the aptamer binding within the whole protein, with a positive cooperative effect (enhanced affinity of a binding site when the neighboring one is occupied). Cooperativity seems to be the responsible for the hardly affected dissociation constant (K_d_) values for the protein-aptamer interaction (Figure 3, panel C), when comparing with those for the 33-mer peptide. Conversely, the chemisorption of the oligonucleotide receptor was revealed to be more deleterious for the subsequent recognition of the 33-mer peptide (Figure 3, panel D). An equivalent approach trying to specifically capture the whole protein was not efficient. This was attributed to the remarkable avidity of the protein for the gold surface because of the multiple sulfur atoms within its structure.

The minor effect on the affinity recorded when 33-mer peptide was immobilized allowed for a study of the influence of the aptamer tagging. When aptamers were labeled with biotin, a 2-fold increase in K_d_ value was measured for Gli1 aptamer, and Gli4 affinity was less affected (Figure 3, panel E), while the incorporation of a fluorescein tag was more disturbing. The fluorescein-modified aptamer affinity towards the immobilized whole protein was two (Gli1) or even three (Gli4) times lower than the ones towards the peptide (Figure 3, panel F). A positive cooperative binding was also operating, which probably allowed for the measurement of the perturbed interaction. 

It is also important to emphasize that, despite being in greater proportion in the enriched pool, Gli1 aptamer is not the most akin aptamer to the target [23,27]. This seemingly contradictory fact is not uncommon and can be attributed to a certain preference in SELEX process for sequences that bind the peptide more rapidly, rather than those with a higher binding affinity. This result is reasonable because the evolution pressure was exerted not only on strength (rinses) but also on time (kinetics).

**Figure 4:**
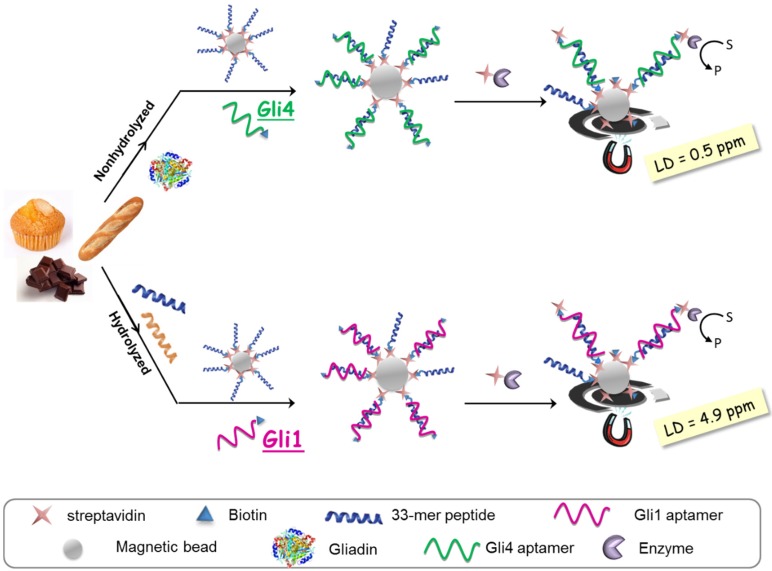
Aptamer-based assays for gliadin determination in processed foods.

The aptassay designed was judiciously guided by the above-mentioned results [28]. The 33-mer peptide was immobilized on magnetic beads to take advantage of the minute impact on the binding affinity, and a biotin-tagged aptamer was used because of its superior performance in comparison to other markers. A competitive assay format was chosen, where competition between the offending target in solution and the attached 33-mer peptide for a limited amount of biotinylated aptamer takes place. The captured aptamer onto the sensing phase is electrochemically quantified using a redox reporter enzyme, which is inversely related to the gluten content in the sample (Figure 4).

As expected, higher sensitivity for Gli4 was obtained on account of its greater affinity using PWG as a gliadin standard [28]. Specifically, it was possible to detect as little as 0.5 ng·L^−1^ of PWG using Gli4 aptamer and 4.9 ng·L^−1^ with Gli1 aptamer even when ethanol, the main component of the extraction solution, is present. Taking into account the compulsory sample extraction step (1:500 sample dilution) and a consensus gliadin/gluten ratio of 1:2 [29], the previous detection limits are translated into 0.5 and 4.9 ppm of gluten for Gli4 and Gli1 aptamers, respectively. Of note, the former is the lowest value achieved so far.

Selectivity studies pointed out that both aptamers, Gli1 and Gli4, recognize wheat, barley, and rye with similar sensitivity and are insensitive to proteins from rice, corn, and soya, widely used in gluten-free products due to their harmlessness to celiac patients. The difference lies in the controversial avenins, which are identified only by Gli4 aptamer, albeit with some lower sensitivity. This means that a combined assay using two aptamers raised from a single SELEX procedure can become a useful and easy tool for rapid and cost-effective discrimination between toxic and controversial sources of gluten. This would provide valuable information for CD patients and their capacity to decide. It is noteworthy that the method internationally accepted by the Codex Alimentarius Commission based on R5 antibody (against ω-secalins) cannot detect avenins, while those immunoassays relied on monoclonal antibodies described against the 33-mer gliadin peptide, G12 and A1, also exhibit a certain affinity for avenins, even though the 33-mer peptide is not included in their structure [30]. Gli1 aptamer showed superior performance when the 33-mer peptide was used as a calibration standard due to an unexpected but consistent microscopic aggregation of beads. Therefore, Gli1 aptamer is recommended for the analysis of hydrolyzed food. 

Another competitive aptamer-based assay, involving G33 aptamer against Sigma gliadin, has been recently developed [26]. In that case, gliadin in solution competes with gliadin bound to a microtiter plate by a G33 aptamer. The amount of oligonucleotide receptor fixed in the well is then eluted and quantified by PCR. The limit of detection was arbitrarily set at 100 ppb of gliadin, even though analytical signals from non-toxic cereals are indistinguishable, or even lower (signal-off in the way proposed). In addition to the complicated scheme, no direct comparison can be made because the equivalence in gluten content is unknown since no treatment protocol for real food samples is proposed. 

Our promising “academic” achievement culminated in an innovative and appealing approach to real-world applications [28]. Blind samples of different origins and unknown gluten content, as well as samples from interlaboratory tests, were analyzed with both the Gli4-based aptassay (the most sensitive to determine non-hydrolyzed gluten) and the Gli1-based aptassay (the choice for hydrolyzed samples). The analyses revealed a good correlation with G12-based and R5-based ELISA commercial methods. Importantly, there were no false-negative results that could have questioned the usefulness of our methods to ensure food security for gluten-sensitive population. The presence of a few “false positives” when compared with the R5 method points to the higher sensitivity of our aptassay. Likewise, the aptamer-based methodology proved to be compatible with both extraction protocols, the conventional one consisting of a separation in 2 M NaCl followed by an extraction in 60% ethanol, and the method using the cocktail solution containing β-mercaptoethanol and guanidine chloride as reducing and disaggregating agents, respectively, to break the interchain S–S bonds and to solubilize gluten followed by an alcoholic extraction. Nonetheless, higher gluten values were systematically recorded when using the cocktail solution, probably because of a larger yield of extraction. Samples containing fats pose special challenges, and, surprisingly, using the same ELISA test certain variability in the results has been reported. This issue still remains with aptassays, meaning that the main challenge lies in target extraction.

According to Codex Alimentarius, food products with a gluten content of less than 20 ppm can be labeled as “gluten-free” [5]. The official method, a sandwich-type ELISA based on R5 antibody, has a limit of detection (LD) of 5 ppm of gluten, while for the same antibody-based competitive assay the LD is 0.75 ppm of gluten. The most sensitive commercial immunoassay, a competitive ELISA based on G12 antibody and marketed by Biomedal, is able to detect as little as 0.6 ppm of gluten. The competitive aptassay developed becomes sufficiently sensitive and selective to be applied to the determination of gluten in foods to assess compliance with labeling legislation and to provide consumers with more information. The Gli4 aptamer-based method would allow for a noticeable lowering of the threshold value in the legislation, protecting more effectively the most vulnerable celiac population. Likewise, the detection limit of the Gli1 aptamer-based assay verifies the compliance with current legislation for gluten-free food labeling.

## 5. Outlook

Methods for detecting gluten in foods have developed significantly in the last decade. However, it is not clear to what extend these methods can ensure the protection of all celiac consumers, taking into account the variability of the gluten safety threshold among individual patients. Hence, it is paramount to continue developing analytical methods that are even more sensitive, additionally meeting the requirements of reliability, accuracy, and low cost. We have described a new kind of receptor (aptamers) for the gluten-derived harmful proteins or peptides that can successfully face some of the remaining challenges of gluten detection. The new receptors present high affinity and binding selectivity. In addition, they can be easily labeled with different reporter molecules at a relatively low production cost. These attributes make aptamers ideal reagents for the development of chemical sensors and analytical assays.

As discussed here, aptamer-based assays are a new generation of methods for gluten quantification. Although still in its infancy, this sensitive technology will undoubtedly continue to advance. It is reasonable to expect that detailed, comprehensive studies to obtain extensive molecular-level information about the aptamer-protein and aptamer-peptides interactions could allow for a better understanding of the structural basis of this highly specific interaction, leading to new modified aptamers with improved affinity properties. In addition, the combination of aptamers with nanomaterials, such as carbon nanotubes, graphene, and metal nanoparticles, either as an immobilization platform or as label agents, and the adaptation of aptamers to a great variety of read-out configurations will likely result in sensitive and easy-to-use tools for gluten detection.

## Figures and Tables

**Table 1:** Commercial enzyme-linked immunosorbent assays for gluten detection in food.

Antibody	Manufacturer	Commercial Name	Format	L.D. (ppm gluten)
Skerrit [18]	Neogen	Biokits	Sandwich	1
	BioCheck	Gluten Check	Sandwich	5
	ELISA technologies	EZ-Gluten	Lateral flow device	10
R5 [19]	Bio Control Systems	TransiaPlateProlamins	Sandwich	3
	Neogen	Veratox	Sandwich	5
	R-Biopharm	RidaScreen	Sandwich	5
		RidaScreenGliadincompetitive	Competitive	5
		Rida Quick	Lateral flow device	5
	Ingenasa	Ingezim gluten	Sandwich	3
G12 [20]	Biomedal	Gluten Tox Sandwich	Sandwich	0.6
		Gluten Tox Competitive	Competitive	3
		Gluten Tox ELISA Sticks	Lateral flow device	3
	RomerLab	Agra Quant	Sandwich	2
		Agra-Strip	Lateral flow device	5
